# Macro- and Micro-Galvanic Corrosion Mechanisms in Symmetric and Asymmetric Double-Sided Friction Stir-Welded 7A65 Aluminum Alloy Joints

**DOI:** 10.3390/ma19153314

**Published:** 2026-08-04

**Authors:** Chen Chen, Yichao Zhu, Zhiping He, Yanfei Wang, Weifeng Xu, Chenyang Qiu, Zhennan Liu

**Affiliations:** 1 China Helicopter Research and Development Institute, Jingdezhen 333001, China; 2 School of Materials Science and Engineering, Northwestern Polytechnical University, Xi’an 710072, China

**Keywords:** double-sided friction stir welding, Al-Zn-Mg-Cu alloy, corrosion, microstructure

## Abstract

The corrosion behavior of symmetric (S-joint) and asymmetric (A-joint) double-sided friction stir-welded (DS-FSW) 7A65 aluminum alloy thick plates was investigated in 3.5 wt% NaCl solution. The S-joint, produced using the same large tool (Φ30 mm) for both passes, imposes two high-heat thermal cycles that result in insufficiently fragmented intermetallic particles (IMPs), coarse grains, and severely overaged heat-affected zones (HAZs). In contrast, the A-joint, employing a smaller tool (Φ24 mm) for the second pass, reduces the total heat input and achieves a refined microstructure with fine (2–3 µm), rounded IMPs in the second-pass weld nugget (WNZ-S) and less degraded HAZs. Electrochemical measurements reveal that the HAZ-Overlap (HAZ-O) is the most anodic zone in both joints. The S-joint shows a larger potential spread (up to ~120 mV) and higher corrosion current density than the A-joint. The hierarchical galvanic coupling, where macro-galvanic corrosion between the anodic HAZ-O and cathodic WNZs drives severe localized attack, while micro-galvanic corrosion around coarse IMPs initiates trenching, is elucidated. The A-joint mitigates this damage due to its reduced galvanic driving force (smaller potential spread of ~74 mV) and improved microstructural homogeneity. The enhanced corrosion resistance of the A-joint is attributed to grain refinement, effective IMP fragmentation, and a less degraded HAZ microstructure.

## 1. Introduction

The Al-Zn-Mg-Cu alloy 7A65, with a high Zn/Mg ratio, offers a favorable combination of strength, toughness and corrosion resistance, making it a promising material for thick-section aerospace components, such as fuselage frames and bulkheads [1,2,3]. For joining plates thicker than 10 mm, double-sided friction stir welding (DS-FSW) has been developed to overcome the limitations of single-pass FSW, including excessive heat input, insufficient penetration, and through-thickness microstructural gradients [4,5,6,7,8,9]. In DS-FSW, two welding passes are applied from opposite sides of the plate. The conventional symmetric DS-FSW (S-DS-FSW) uses identical tools for both passes, whereas asymmetric DS-FSW (A-DS-FSW) employs a smaller or differently designed tool for the second pass, aiming to reduce the thermal impact on the first weld [8,10,11]. Crucially, the tool size, specifically the shoulder diameter and pin length, directly determines the frictional heat generation and plastic strain imposed on the material. Tool 1 (shoulder: Φ30 mm; pin length: 16 mm) generates significantly higher peak temperatures and a wider thermomechanically affected zone compared to Tool 2 (shoulder: Φ24 mm; pin length: 12 mm). Thus, the S-DS-FSW joint (hereafter referred to as the S-joint) imposes two high-heat thermal cycles, while the A-DS-FSW joint (A-joint) reduces the total heat input, leading to distinct microstructural evolutions and corrosion responses. Although previous studies have compared the microstructures and mechanical properties of S- and A-DS-FSW joints [11], their corrosion behaviors have received little attention.

For high-strength aluminum alloys in chloride-containing service environments (e.g., marine or coastal atmospheric conditions), corrosion resistance is as critical as mechanical integrity [2,12,13]. In DS-FSW joints, the dual-pass thermal cycle inevitably introduces complex microstructural heterogeneity along both the transverse and thickness directions. Key microstructural zones include the weld nugget, thermomechanically affected zone, heat-affected zone (HAZ), and double-stirred zone (DSZ), where the merging of the upper and lower welds is unique to DS-FSW. These regions differ significantly in terms of their precipitate sizes/distributions, grain structures, dislocation densities, and residual stresses, leading to spatially varying electrochemical activity [14,15,16]. Under immersion in NaCl solution, such heterogeneity often drives localized corrosion, where certain zones become preferential anodic sites while others act as cathodes. Furthermore, the coupling between different zones across the weld cross section can result in differential corrosion behavior that evolves with the immersion time [17,18,19].

Despite the practical importance, the current understanding of the corrosion behavior of thick-plate DS-FSW joints remains very limited. To the best of our knowledge, no systematic study has addressed the local electrochemical corrosion behavior of the bond zone and HAZ in DS-FSW 7A65 joints, nor has the overall corrosion evolution of the entire weld cross section been characterized under prolonged immersion. In particular, the influence of welding asymmetry (S- vs. A-DS-FSW) on the localized corrosion susceptibility and time-dependent corrosion morphology across the joint has not been explored. Such understanding is essential for assessing the long-term durability of DS-FSW components and for optimizing welding parameters to balance mechanical and corrosion performance.

We hypothesize that the asymmetric DS-FSW configuration, by employing a smaller tool for the second pass, reduces the total heat input, refines the IMPs, and mitigates HAZ degradation, thereby reducing the galvanic driving force and enhancing corrosion resistance compared to the symmetric configuration. To test this hypothesis, this work systematically investigates the corrosion behavior of S-DS-FSW and A-DS-FSW joints of 7A65 aluminum alloy. The study focuses on two aspects: (i) the localized electrochemical corrosion behavior of individual microstructural zones, with an emphasis on the WNZ-DSZ, the HAZ from the upper and lower welds, as well as the HAZ-Overlap, and (ii) the overall corrosion evolution of the weld cross section during immersion in 3.5 wt% NaCl solution for durations of 2 to 10 h. The experimental parameters and materials are identical to those used in a companion study on mechanical properties [11], enabling a direct correlation between corrosion resistance and microstructural features. By comparing the two DS-FSW configurations, this work aims to identify the configuration that offers superior corrosion resistance while maintaining mechanical integrity, thereby providing practical guidance for the manufacture of thick-plate aerospace structures.

## 2. Experiment

### 2.1. Materials

The base material (BM) used in this investigation was a 27 mm thick 7A65-T7451 plate. The nominal chemical composition of the alloy is listed in Table 1, and its initial microstructure is detailed in our previous work [11]. DS-FSW was conducted on an FSW-RL31-010 machine. The welding parameters were fixed at a rotational speed of 300 rpm and a traversing speed of 120 mm/min. After completing the first weld, surface flash was removed by gentle polishing, and the plate was then flipped over to perform the second weld. The welding direction remained the same for both passes.

**Figure 1:**
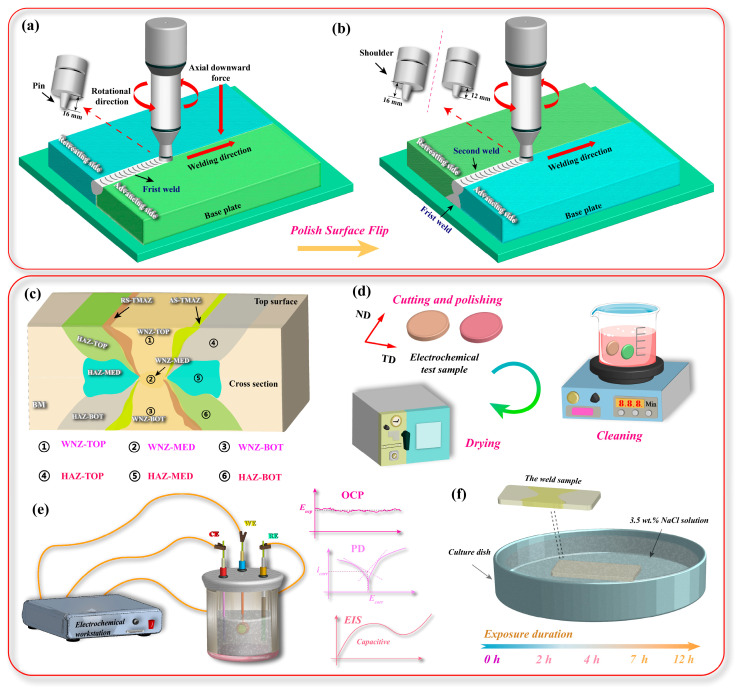
Schematic illustrations of (**a**,**b**) the welding processes for the S-joint and A-joint, (**c**) the cross-sectional zones of the weld, (**d**) the preparation procedure for electrochemical specimens, (**e**) the electrochemical test setup, and (**f**) the whole-weld immersion test arrangement.

Two welding tools, both made of H13 steel, were employed. Tool 1 had a shoulder diameter of 30 mm and a pin length of 16 mm; Tool 2 had a shoulder diameter of 24 mm and a pin length of 12 mm. Both tools featured a tapered, threaded pin with three flats. For the S-DS-FSW joint (hereafter referred to as the S-joint), Tool 1 was used for both the first and second welds. For the A-DS-FSW joint (A-joint), Tool 1 was applied in the first weld and Tool 2 in the second weld. The schematic diagram is shown in Figure 1a. To prepare metallographic samples, the welded plates were cross-sectioned, ground, and polished. The polishing procedure consisted of sequential grinding with SiC papers up to 4000 grit, followed by diamond paste polishing (1 µm) and final polishing with a colloidal silica suspension (0.05 µm) to achieve a scratch-free surface. The polished cross sections were then etched with Keller’s reagent (2 mL HF + 3 mL HCl + 5 mL HNO_3_ + 190 mL H_2_O) for 15–20 s to reveal the microstructural features. Grain size measurement was performed on the optical microscope (OM, OLYMPUS GX71, Olympus Corporation, Tokyo, Japan) images using the linear intercept method in accordance with ASTM E112. For each characteristic zone, at least five randomly selected micrographs were analyzed. On each image, three to four straight test lines were superimposed, and the number of grain-boundary intercepts was counted. The mean lineal intercept length was calculated as the total test line length divided by the number of intercepts, with a minimum of 50 intercepts counted per zone. The average grain size for each zone was then determined from the mean intercept values across all measured images. Microstructural observation was performed using a field-emission scanning electron microscope (FE-SEM) (FEI Helios G4CX; FEI Company, Hillsboro, OR, USA) equipped with an energy-dispersive X-ray spectroscopy (EDS) probe (X-Max 80; Oxford Instruments, Abingdon, UK). Figure 1c schematically illustrates the six characteristic zones examined in this work for both the S-joint and A-joint, namely, the first-pass WNZ (WNZ-F), second-pass WNZ (WNZ-S), double-stirred zone (DSZ), first-pass HAZ (HAZ-F), second-pass HAZ (HAZ-S), and overlap HAZ (HAZ-O).

### 2.2. Electrochemical and Immersion Corrosion Testing

All electrochemical measurements were carried out in a 3.5 wt% NaCl solution at ambient temperature using an IVIUM Vertex.C.EIS workstation. A conventional three-electrode cell was employed, consisting of a Ag/AgCl (saturated KCl) reference electrode, a platinum mesh counter electrode, and the working electrode. The working electrode was prepared from selected regions of the DS-FSW joints (e.g., the overlap zone or the HAZ of the upper/lower weld) with an exposed geometric area of 1.5 cm^2^. Before each measurement, the working electrode was immersed in the electrolyte for 1 h to attain a stable open-circuit potential (OCP). Electrochemical impedance spectroscopy (EIS) was then performed at the OCP over a frequency range from 10^5^ Hz down to 10^−2^ Hz, with a sinusoidal AC amplitude of 10 mV. Subsequently, potentiodynamic polarization (PD) scans were conducted from a cathodic potential of −0.9 V (vs. Ag/AgCl) toward the anodic direction at a sweep rate of 0.167 mV/s until the anodic current density exceeded 1 mA/cm^2^. To ensure data reliability, each electrochemical test was repeated on at least three independently prepared samples. The corresponding schematic diagram is shown in Figure 1e.

Immersion tests were performed on specimens that comprised the entire cross section of the DS-FSW joints, allowing for observation of the overall corrosion evolution. According to the ASTM G31-72 standard, the volume of the 3.5 wt% NaCl solution was controlled to maintain a ratio of electrolyte volume to exposed specimen area of 20:1 (mL:cm^2^), as illustrated in Figure 1f. The specimens were immersed for durations ranging from 2 to 10 h, with intermediate extraction at 2, 4, 7, and 10 h. After each designated immersion period, the samples were removed, gently rinsed with deionized water, dehydrated in ethanol, and dried in a vacuum desiccator. The corroded surfaces were then documented under identical illumination and magnification conditions for subsequent analysis. All immersion tests were carried out in duplicate to confirm reproducibility. After each corrosion test, the microstructural corrosion morphology was characterized using a field-emission scanning electron microscope (FE-SEM) (FEI Helios G4CX) equipped with an energy-dispersive X-ray spectroscopy (EDS) probe.

## 3. Results

### 3.1. Microstructures

**Figure 2:**
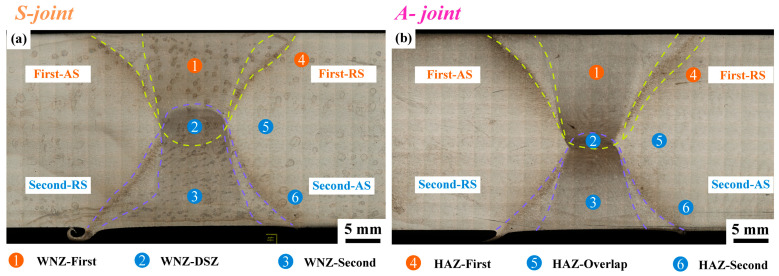
Cross-sectional macrostructures of (**a**) S-joint and (**b**) A-joint.

Figure 2 presents the cross-sectional macrostructures of the S-joint and A-joint. Both joints exhibit full penetration without voids or unwelded regions, especially in the central overlap zone where the two welds meet. The cross sections reveal a characteristic “dumbbell” profile. The WNZs swell near the top and bottom surfaces yet taper progressively toward the mid-thickness. This morphology arises from the combined thermomechanical action of the two passes. For the S-joint (Figure 2a), the same tool (Tool 1) used for both passes yields nearly symmetric upper and lower welds, with comparable WNZ widths and cross-sectional areas. The boundary between the two WNZs is diffuse, and the DSZ appears slightly darker in contrast due to additional stirring and grain refinement. In contrast, the A-joint (Figure 2b) exhibits marked asymmetry: the upper weld (Tool 1) produces a broader WNZ and a wider thermomechanically affected zone (TMAZ) than the lower weld (Tool 2). Quantitative measurements show that the WNZ width at mid-height is approximately 12.6 mm for the upper weld versus 9.8 mm for the lower weld. The DSZ in the A-joint is notably narrower and more sharply delineated, reflecting the reduced stirring volume of Tool 2.

Another notable feature is the HAZ, which appears as a lightly etched band flanking both sides of the TMAZ. In both joints, the HAZ extends further into the base metal on the advancing side (AS) than on the retreating side (RS). However, the HAZ width differs between the upper and lower welds in the A-joint: the upper-weld HAZ is slightly broader, consistent with the higher heat input from the larger tool. Additionally, fine and arc-shaped material flow lines (often referred to as “onion rings”) are visible within each WNZ, indicating successive material transfer during the rotation and translation of the tool. These flow lines are more distinct in the S-joint, where the larger tool generates more pronounced mixing. The RS of the second weld in the S-joint exhibits a larger flash than that of the RS of the second weld in the A-joint, in agreement with the surface observations.

**Figure 3:**
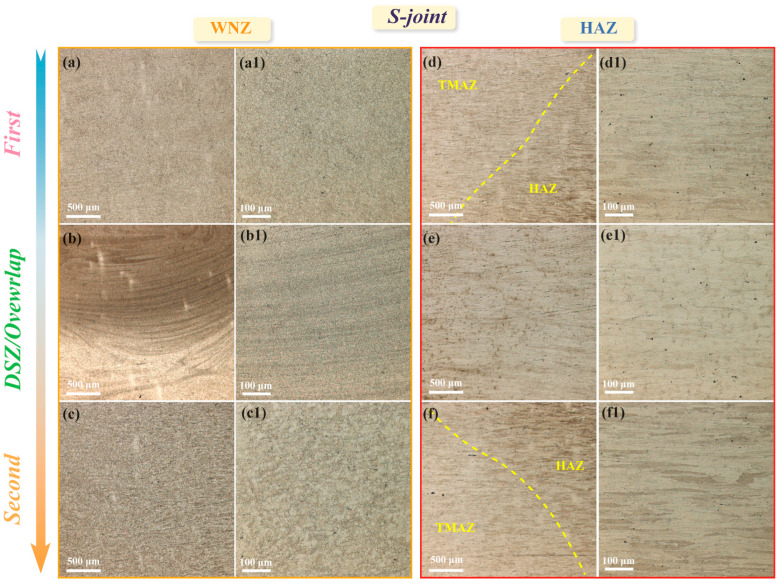
Microstructures of S-joint: (**a**–**c**) WNZ-F, DSZ, and WNZ-S; (**d**–**f**) HAZ-F, HAZ-O, and HAZ-S. (**a1**–**f1**) Corresponding higher-magnification images.

**Figure 4:**
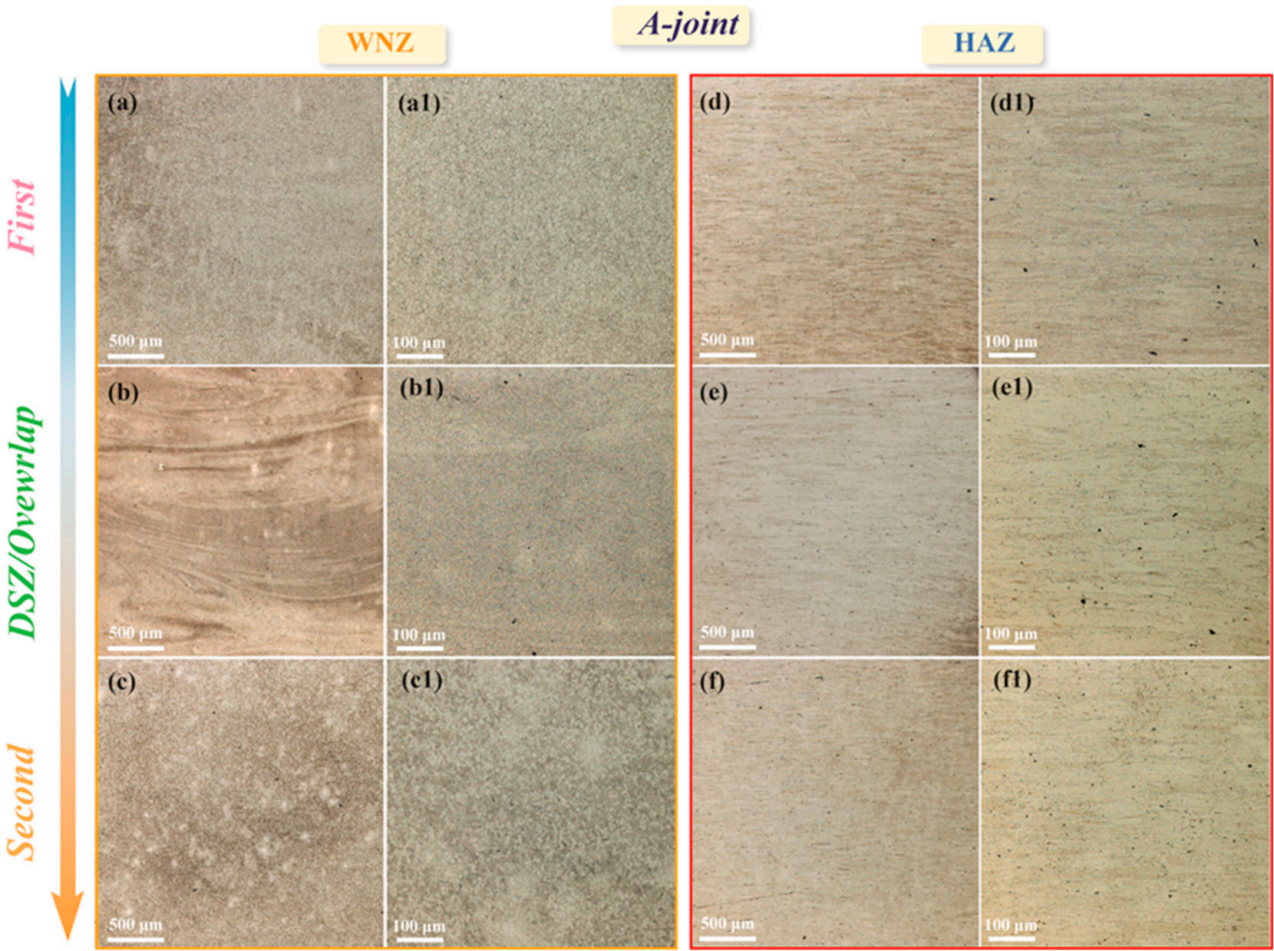
Microstructures of A-joint: (**a**–**c**) WNZ-F, DSZ, and WNZ-S; (**d**–**f**) HAZ-F, HAZ-O, and HAZ-S. (**a1**–**f1**) Corresponding higher-magnification images.

Figure 3 and Figure 4 present the optical microstructures of the six characteristic zones of the S-joint and A-joint. In the S-joint, the upper and lower welds exhibit nearly mirror-symmetric morphologies (Figure 3a,c). The two WNZs consist of fully recrystallized equiaxed grains with an average size of 5–15 µm. The HAZs on both sides exhibit elongated or slightly elongated non-recrystallized grains (Figure 3d–f). The TMAZ displays bent, elongated grains that are similarly deformed on each side. The DSZ shows a moderately refined structure (average grain size: 3–5 µm) with a diffuse boundary and darker etching contrast, indicating two-pass stirring (Figure 3b).

In contrast, the A-joint exhibits pronounced microstructural asymmetry. The upper WNZ is coarser (8–12 µm) because the larger tool generates higher frictional heat and greater plastic strain, whereas the lower WNZ is finer owing to the reduced heat input of Tool 2 (Figure 4a,c). The HAZ on the upper side is broader, while that on the lower side is narrower [8,20]. Notably, the DSZ in the A-joint exhibits a significantly refined grain structure, with an average grain size of approximately 2 µm. This refinement is attributed to the additional plastic deformation imposed by the smaller tool during the second pass, which provides further stirring without causing excessive grain coarsening. Moreover, the boundary between the DSZ and the surrounding WNZ is more sharply delineated in the A-joint than in the S-joint. (Figure 4b). These microstructural differences directly stem from the asymmetric thermomechanical cycles imposed by the two welding tools. Specifically, the larger tool generates higher peak temperatures and greater plastic strain, promoting coarser recrystallization and a wider HAZ. The smaller tool introduces less heat but adds further deformation, leading to grain refinement, particularly in the overlapping region.

### 3.2. The Intermetallic Particles (IMPs)

**Figure 5:**
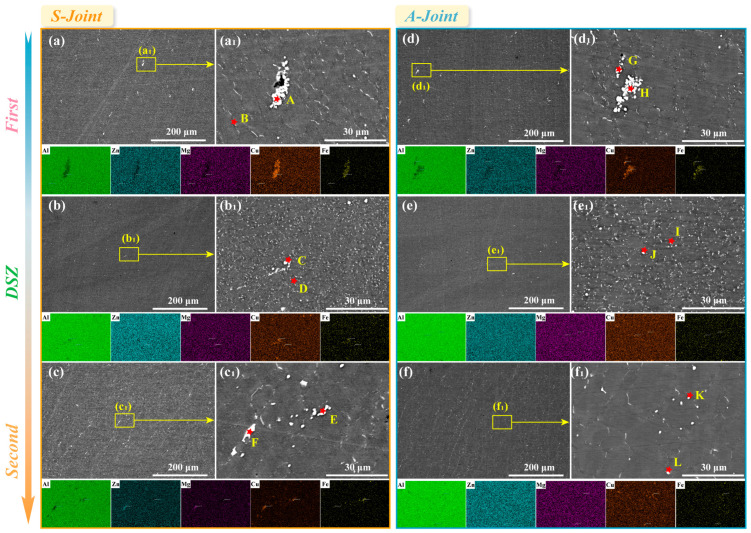
SEM images showing the distribution and morphology of the IMPs in the S-joint ((**a**–**c**): WNZ-F, DSZ, WNZ-S) and A-joint ((**d**–**f**): WNZ-F, DSZ, WNZ-S). Figures (**a1**–**f1**) are the corresponding higher-magnification SEM images together with the EDS mapping results.

Figure 5 presents SEM images of the IMPs in the WNZ-Fs, DSZs, and WNZ-Ss of the S- joint and A-joint. The low-magnification images (a–c for the S-joint and d–f for the A-joint) and high-magnification counterparts (a1–c1; d1–f1) reveal the distribution and morphology of the coarse particles, identified by EDS as Al-Cu-Fe and Al-Zn-Mg-Cu particles (points “A”-“l”, Table 2).

In the S-joint, the WNZ-F and WNZ-S show insufficient fragmentation, and some large particles of 15–30 µm are observed under low magnification (Figure 5a,c). High-magnification images further reveal local agglomerations of coarse particles and pronounced precipitation along grain boundaries, with both passes exhibiting nearly identical characteristics. In the DSZ, intense stirring from both passes extensively fragments the IMPs to nanoscale dimensions (mostly <1 µm), resulting in a dense distribution of fine particles that gives rise to the bright white contrast visible throughout the image field. (Figure 5b). For the A-joint, a distinct asymmetry emerges. The WNZ-F resembles that of the S-joint, with insufficient fragmentation, featuring large particles up to ~25 µm and local clusters of finer fragments under high magnification (Figure 5d). In contrast, the WNZ-S exhibits much more effective particle breakup. It shows a chain-like distribution at low magnification and uniformly dispersed, rounded particles of only 2–3 µm at high magnification (Figure 5f). This indicates that the smaller tool promotes the efficient fragmentation and spheroidization of the brittle Al-Cu-Fe and Al-Zn-Mg-Cu particles. In the DSZ of the A-joint, similarly intense fragmentation produces nanoscale particles with a dense distribution comparable to that of the DSZ in the S-joint (Figure 5b,e). However, the DSZ in the A-joint contains a significantly lower proportion of larger residual particles (around 2 µm) than the DSZ in the S-joint, suggesting that sequential stirring by the larger tool followed by the smaller tool achieves more thorough refinement.

In summary, the S-joint displays symmetric but less extensive particle breakup in the two WNZs, with large residual particles and grain-boundary precipitates, while the A-joint yields a gradient (coarse first-pass WNZ vs. fine, rounded, uniform second-pass WNZ). Both joints exhibit nanoscale fragmentation in the DSZ, with the A-joint showing a higher refinement degree and fewer coarser remnants.

**Figure 6:**
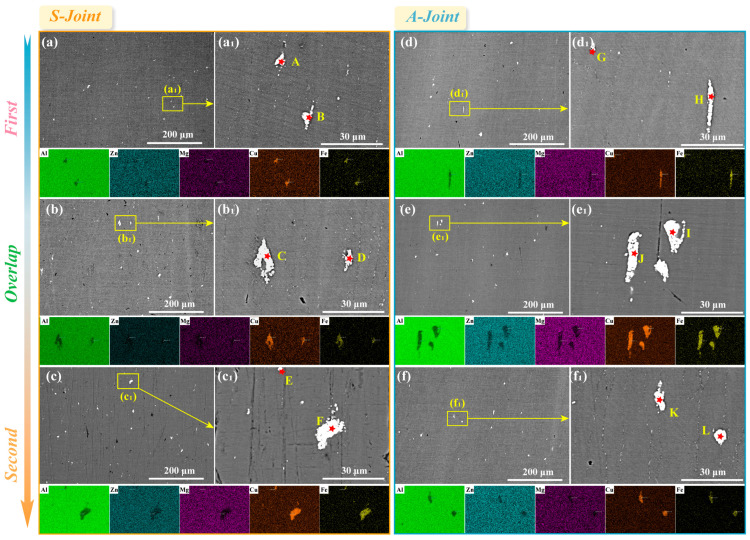
SEM images showing distribution and morphology of IMPs in S-joint ((**a**–**c**): HAZ-F, HAZ-Overlap, HAZ-S) and A-joint ((**d**–**f**): HAZ-F, HAZ-Overlap, HAZ-S). Panels (**a1**–**f1**) are corresponding higher-magnification SEM images together with EDS mapping results.

Figure 6 presents the SEM and EDS images of the IMPs in the HAZ-F, HAZ-S, and HAZ-O for the S-joint and A-joint. The EDS point scan results confirm that these particles are primarily Al-Cu-Fe and Al-Zn-Mg-Cu particles (points “A”-“l”, Table 3).

Compared to the WNZs, all HAZ regions exhibit notably larger volume fractions and coarser particle sizes, typically ranging from 10 µm to 30 µm, due to the absence of intense mechanical stirring. In the S-joint, the HAZ-F and HAZ-S show that poorly fragmented Al-Cu-Fe and Al-Zn-Mg-Cu particles are dispersed throughout the HAZ matrix, with occasional clustering, and the grain boundaries contain a high density of fine precipitates (Figure 6a,c). In the HAZ-O of the S-joint, which experiences the thermal cycles of both passes, grain-boundary precipitation becomes even more pronounced (Figure 6b). For the A-joint, a clear asymmetry emerges, and the HAZ-F resembles that of the S-joint, with coarse (10–30 µm), blocky particles and moderate grain-boundary precipitation (Figure 6d). In contrast, the HAZ-S contains somewhat finer and more rounded particles (mostly 8–20 µm), likely due to the lower peak temperature of the smaller tool (Figure 6f). The HAZ-O of the A-joint shows the most extensive grain-boundary precipitation among all examined regions, with dense precipitate arrays lining the boundaries (Figure 6e). The Al-Zn-Mg-Cu and coarse Al-Cu-Fe particles remain similar in size to those in the HAZ-O of the S-joint but exhibit a more heterogeneous distribution. Overall, both joints share the common HAZ feature of coarse, poorly fragmented Al-Cu-Fe and Al-Zn-Mg-Cu particles and significant grain-boundary precipitation.

To quantitatively distinguish the critical microstructural differences between the S-joint and A-joint, the grain size, IMP characteristics, and grain-boundary precipitation in the HAZ regions of both joints are summarized in Table 4. The A-joint exhibits finer grains in the HAZ-S (8–15 µm) compared to the S-joint HAZ-S (10–20 µm), attributed to the lower heat input from the smaller tool. More importantly, the IMPs in the A-joint HAZ-S are finer (8–20 µm) and more rounded than their S-joint counterparts (10–30 µm, angular), indicating reduced thermal coarsening. The grain-boundary precipitation is also less severe in the A-joint HAZ-S. These microstructural refinements directly contribute to the improved corrosion resistance of the A-joint, as discussed in Section 4.

The microstructural refinement in the A-joint, particularly the finer grains in the HAZ-S and the more homogeneous IMP distribution, is consistent with the higher and more uniformly distributed hardness reported in our companion study on the mechanical properties of these joints [11]. The improved hardness in the A-joint (Asy-DS-FSW) compared to the S-joint (Sym-DS-FSW) further corroborates the less severe thermal degradation of the HAZ, which directly contributed to the enhanced corrosion resistance observed in the present work.

Overall, both joints share the common HAZ feature of coarse, poorly fragmented Al-Cu-Fe and Al-Zn-Mg-Cu particles and significant grain-boundary precipitation.

### 3.3. Electrochemical Corrosion Behaviors

#### 3.3.1. The OCP Evolution

The OCP of each microstructural zone was monitored for 60 min in 3.5 wt% NaCl solution, and the average values over the final 30 min were calculated to compare the steady-state electrochemical activity. Table 5 summarizes the average OCPs for the BM and the six regions of the S-joint and A-joint.

**Figure 7:**
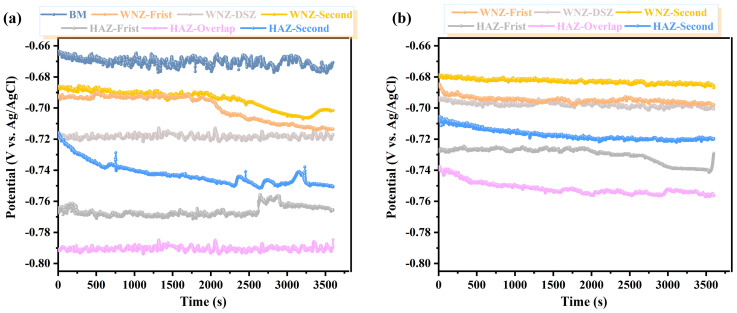
OCP evolution over time for different zones of (**a**) S-joint and (**b**) A-joint.

For the S-joint, the BM exhibits the noblest potential (−0.671 V). The WNZ regions show more negative potentials, indicating higher anodic activity: WNZ-F (−0.712 V), WNZ-S (−0.706 V), and WNZ-DSZ (−0.719 V). The HAZ regions are even more active, with HAZ-F (−0.766 V), HAZ-S (−0.750 V), and HAZ-O (−0.791 V) being the most negative (Figure 7a). Notably, the OCPs of the WNZ-F and WNZ-S initially stabilized around −0.69 V but began to decline after approximately 2000 s, suggesting a time-dependent degradation of corrosion resistance, possibly due to the progressive breakdown of passive films or localized activation [21,22]. In contrast, the WNZ-DSZ exhibited a remarkably stable OCP throughout the measurement, implying a more uniform and stable passive surface. All HAZ regions show a continuous downward drift of the OCP with time, reflecting ongoing activation and increasing susceptibility to corrosion.

For the A-joint, a similar trend in the relative order of potentials is observed, but the absolute potential differences between regions are substantially smaller than those in the S-joint (Figure 7b). The WNZ-F (−0.692 V), WNZ-S (−0.680 V), and WNZ-DSZ (−0.695 V) are again more negative than the BM, while the HAZ regions (−0.731 V for HAZ-F, −0.720 V for HAZ-S, and −0.754 V for HAZ-O) are the most active. Compared with the S-joint, the A-joint shows a narrower spread between the noblest and most active zones, indicating a reduced galvanic driving force across the weld cross section. The OCP of the WNZ-S is slightly less negative than that of the WNZ-F, consistent with its finer and more uniform particle distribution observed in the SEM. The HAZ regions in the A-joint also exhibit a continuous decrease in the OCP over time, similar to the Sym case, but with less pronounced overall drift.

#### 3.3.2. The PD Behaviors

**Figure 8:**
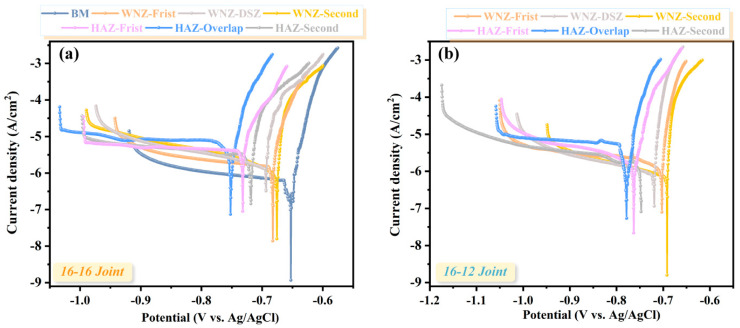
PDP curves of various zones in (**a**) S-joint and (**b**) A-joint.

Potentiodynamic polarization tests were conducted in 3.5 wt% NaCl solution to evaluate the corrosion resistance of individual microstructural zones, as shown in Figure 8. The corrosion potential (*E_corr_*) and corrosion current density (*i_corr_*) obtained by Tafel extrapolation are summarized in Table 6 for the BM and the six regions of the S-joint and A-joint [23,24].

For the S-joint, the BM exhibits the noblest *E_corr_* (−0.652 V) and the lowest *i_corr_* (0.776 μA/cm^2^), indicating the best corrosion resistance (Figure 8a). Among the welded zones, the WNZ regions show moderately negative potentials (WNZ-F: −0.682 V; WNZ-S: −0.676 V; WNZ-DSZ: −0.694 V) and relatively low current densities (1.98, 1.45, and 1.74 μA/cm^2^, respectively), which is consistent with their fine recrystallized grains and fragmented IMPs that promote a more stable passive film. However, the slightly higher *i_corr_* of the WNZ-F compared to that of the WNZ-S may be related to the insufficient fragmentation of Al-Cu-Fe and Al-Zn-Mg-Cu particles and local agglomerations observed in the WNZ-F. The HAZ regions of the S-joint are the most anodically active, with the HAZ-F (−0.746 V, 5.02 μA/cm^2^), the HAZ-S (−0.723 V, 4.41 μA/cm^2^), and especially the HAZ-O (−0.776 V, 7.28 μA/cm^2^) exhibiting the highest corrosion currents. This severe degradation is attributed to the coarse, poorly fragmented Al-Cu-Fe particles (10–30 µm), the dense grain-boundary precipitation, and the overaged precipitate-free zones that serve as preferential sites for anodic dissolution [25,26].

In the A-joint, a similar relative order of the *E_corr_* and *i_corr_* is observed, but importantly, all welded zones show improved corrosion resistance compared to the S-joint (Figure 8b). For the WNZ regions, the A-joint gives slightly less negative potentials and lower current densities (WNZ-F: −0.687 V, 1.79 μA/cm^2^; WNZ-S: −0.678 V, 1.36 μA/cm^2^; WNZ-DSZ: −0.701 V, 1.52 μA/cm^2^), with the most striking improvement occurring in the WNZ-S, where the Al-Cu-Fe and Al-Zn-Mg-Cu particles are uniformly dispersed, rounded, and only 2-3 µm in size. This can reduce cathodic activity and promote a more protective passive layer. The HAZ regions of the A-joint also show markedly lower *i_corr_* values (HAZ-F: 4.47 μA/cm^2^; HAZ-S: 2.56 μA/cm^2^; HAZ-O: 5.89 μA/cm^2^) compared with the S-joint, especially for the HAZ-S (a reduction from 4.41 to 2.56 μA/cm^2^) and HAZ-O (from 7.28 to 5.89 μA/cm^2^). This enhancement is speculated to originate from the lower peak temperature imparted by the smaller tool, which mitigates the thermal coarsening of grain-boundary precipitates in the HAZ, thereby decreasing the number of active anodic sites [27]. Furthermore, the narrower variations in the *E_corr_* and *i_corr_* across the A-joint suggest a more homogeneous electrochemical response, which is expected to lower the driving force for macro-galvanic corrosion between adjacent zones (Figure 8b). The A-joint exhibits a consistently superior performance, particularly in the second-pass WNZ and HAZ. This demonstrates that asymmetric double-sided FSW can effectively enhance the local corrosion resistance of thick-plate AA7A65 joints by refining IMPs and preserving a less degraded HAZ microstructure.

#### 3.3.3. EIS Analysis

**Figure 9:**
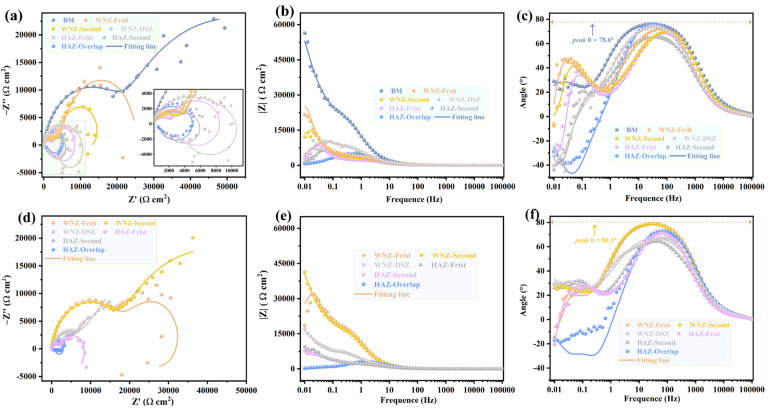
EIS results for the different zones of the S-joint (**a**–**c**) and A-joint (**d**–**f**): (**a**,**d**) Nyquist plots; (**b**,**e**) Bode modulus plots; (**c**,**f**) phase-angle plots.

EIS testing was performed in 3.5 wt% NaCl solution to characterize the corrosion behavior of the distinct microstructural zones of the S-joint and A-joint, as shown in Figure 9, and the corresponding EIS fitting results are summarized in Table 7 and Table 8, respectively.

**Figure 10:**
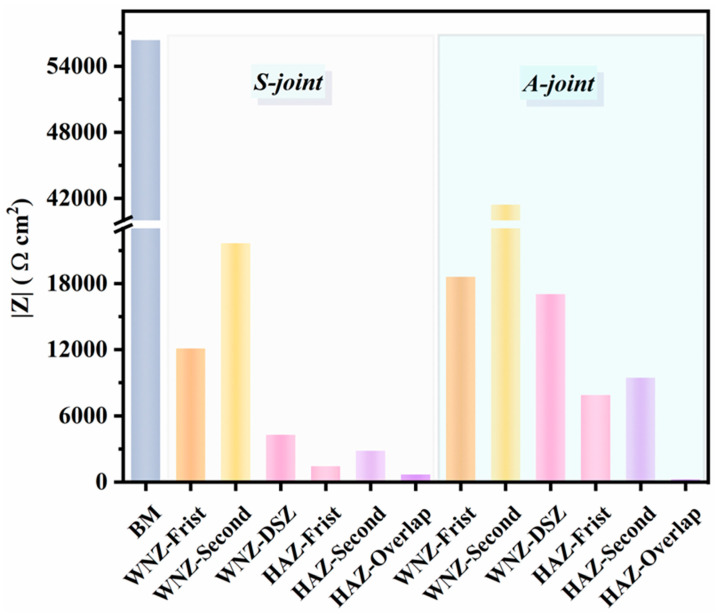
A statistical comparison of the low-frequency impedance moduli (|*Z*|*_mod_*) for different zones of the S-joint and A-joint.

In the S-joint, the Nyquist plot (Figure 9a) reveals that the BM exhibits the largest capacitive arc, which consists of a high-frequency (HF) and low-frequency (LF) capacitive arc, indicating a highly protective passive film and a stable electrochemical interface [28,29]. For all welded zones (WNZ-F; WNZ-S; WNZ-DSZ; HAZ-F; HAZ-S; HAZ-O), a prominent inductive loop appears in the LF region (0.1–0.01 Hz), which is characteristic of localized corrosion initiation, such as pitting or adsorption of chloride ions [30]. The Bode modulus plots (Figure 9b) show the total impedance at low frequency (|*Z*|*_mod_*) for each region: BM (56,370.5 Ω·cm^2^) > WNZ-S (21,664.3 Ω·cm^2^) > WNZ-F (12,112.2 Ω·cm^2^) > HAZ-S (2813.8 Ω·cm^2^) > WNZ-DSZ (4279.4 Ω·cm^2^) > HAZ-F (1410.2 Ω·cm^2^) > HAZ-O (678.7 Ω·cm^2^). The corresponding statistical summary of the impedance is presented in Figure 10. The notably high impedance of the WNZ-S relative to the WNZ-F suggests that the second weld in the symmetric configuration, despite identical tools, produces a slightly more compact passive layer, possibly due to differences in the thermal histories or surface conditions. The DSZ shows an intermediate impedance (4279.4 Ω·cm^2^), lower than both WNZs, which may be attributed to the ultra-fine IMPs that increase the number of active sites for chloride attack [31]. The HAZ regions exhibit severely reduced impedance, especially the HAZ-O (only 678.7 Ω·cm^2^), consistent with their coarse Al-Cu-Fe particles and dense grain-boundary precipitates, which facilitate rapid charge transfer and localized dissolution [32]. The phase-angle plots (Figure 9c) for the BM display a broad, high-angle plateau (close to −80°) over a wide frequency range, indicating nearly ideal capacitive behavior. In contrast, all welded zones show a depressed phase-angle maximum at intermediate frequencies and a sharp decline at low frequencies, often dropping to near 0° or positive angles, which confirms the presence of an inductive loop. This transition is particularly pronounced for the HAZ-O, reflecting its extremely poor corrosion resistance.

For the A-joint, the Nyquist plot (Figure 9d) reveals a different trend. Among the welded zones, the WNZ-S exhibits a relatively large capacitive arc, though it is still smaller than that of the BM. Notably, both the WNZ-S and HAZ-S show no distinct inductive loop, instead presenting a well-defined HF and LF capacitive arc, indicating a more stable passive film and suppressed pitting initiation. In contrast, the WNZ-F, WNZ-DSZ, HAZ-F, and HAZ-O all display a prominent LF inductive loop, similar to the S-joint. The Bode modulus values (Figure 9e and Figure 10) are as follows: WNZ-S (41,411.9 Ω·cm^2^) > WNZ-F (18,612.4 Ω·cm^2^) > WNZ-DSZ (17,027.5 Ω·cm^2^) > HAZ-S (9457.9 Ω·cm^2^) > HAZ-F (7871.6 Ω·cm^2^) > HAZ-O (227.7 Ω·cm^2^). The outstandingly high impedance of the WNZ-S (nearly twice that of the WNZ-F) directly correlates with the effective fragmentation and uniform distribution of fine, rounded Al-Cu-Fe particles observed in the A-joint. This promotes a denser and more protective passive layer. The HAZ-S also shows a relatively high impedance (9457.9 Ω·cm^2^), significantly higher than the HAZ-F (7871.6 Ω·cm^2^) and much higher than the corresponding HAZ-S in the S-joint (2813.8 Ω·cm^2^). This improvement is attributed to the lower heat input of the smaller tool, which mitigates the overaging of grain-boundary precipitates and reduces the density of anodic sites. However, the HAZ-O in the A-joint shows an extremely low impedance (227.7 Ω·cm^2^), suggesting that the combination of thermal cycles from two asymmetric passes may locally intensify grain-boundary sensitization or precipitate coarsening in the overlapping HAZ region. The presence of the inductive loop in the low-frequency region (0.1–0.01 Hz) is characteristic of localized corrosion initiation, such as pitting or adsorption of chloride ions [24]. This inductive behavior indicates that the passive film is locally disrupted, leading to anodic dissolution that is not fully compensated by the film’s repassivation. In the A-joint, the WNZ-S and HAZ-S exhibit no distinct inductive loop, indicating a more stable passive film with suppressed pitting initiation. The phase-angle plots (Figure 9f) show that the WNZ-S and HAZ-S maintain a relatively high phase angle over a broader frequency range, while the WNZ-F, the WNZ-DSZ, and especially the HAZ-O exhibit a sharp phase-angle drop at low frequencies, confirming the inductive behavior.

**Figure 11:**
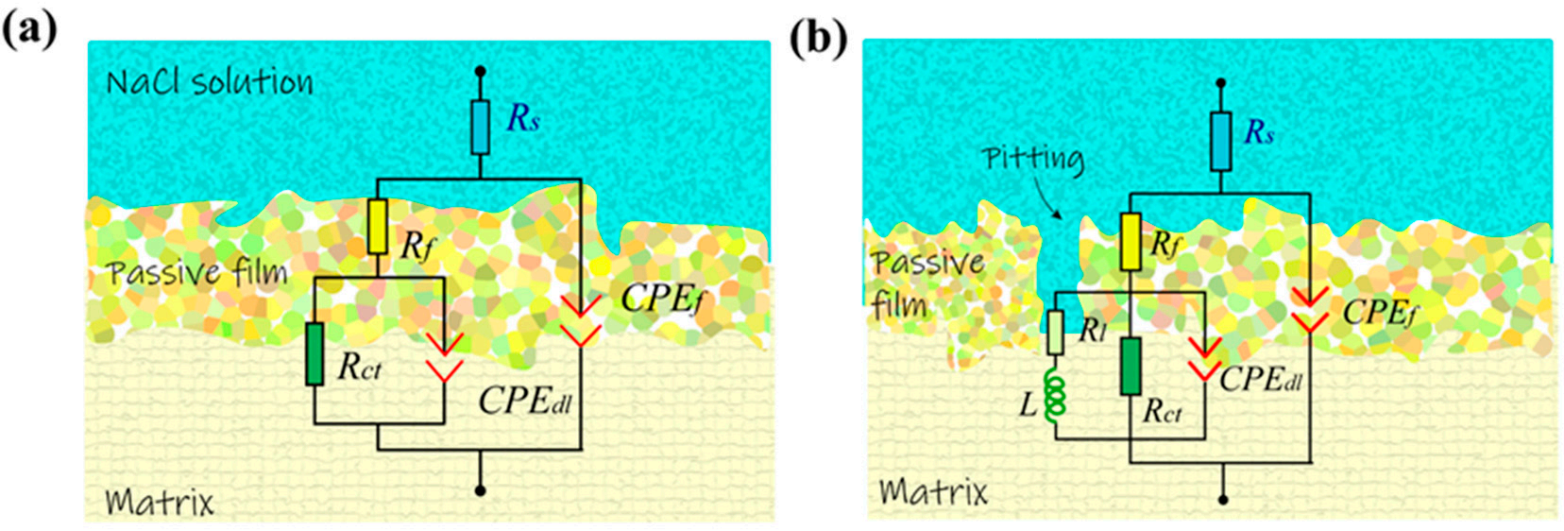
Equivalent circuit models employed for fitting EIS data: (**a**) *R(Q(R(QR)))* and (**b**) *R(Q(R(QR(LR))))*.

To quantitatively interpret the EIS responses, an equivalent circuit model of *R(Q(R(QR)))* was used for regions exhibiting only capacitive loops (the BM, and for the A-joint, also the WNZ-S and HAZ-S), as shown in Figure 11a. *R_s_* is the solution resistance, *Q* is the constant-phase element for the passive film, and *(R_f_(Q_dl_R_ct_))* represents the film resistance, double-layer capacitance, and charge transfer resistance. The equivalent circuit model (*R(Q(R(QR)))*) used for regions exhibiting only capacitive loops (the BM, and for the A-joint, also the WNZ-S and HAZ-S) confirms that the corrosion process is dominated by charge transfer, not diffusion. The absence of a Warburg element indicates that the mass transport of ions in the electrolyte is not the rate-limiting step; rather, the kinetics of the anodic and cathodic reactions control the corrosion rate. For all regions showing an LF inductive loop (most welded zones in both joints), a modified circuit (*R(Q(R(QR(LR))))*) was employed (Figure 11b), where an *L* (inductance) in series with an *R_l_* (inductive resistance) is added to account for the relaxation of adsorbed intermediates or the onset of pitting. The presence of the inductive loop indicates that the passive film is locally disrupted, leading to anodic dissolution that is not fully compensated by the film’s repassivation [33,34]. In the A-joint, the WNZ-S and HAZ-S exhibit much higher impedance and no inductive loop. This indicates that the asymmetric process with a smaller tool enhances local corrosion resistance by refining IMPs and preserving a more intact passive film. However, the HAZ-O remains critically vulnerable.

### 3.4. Immersion Corrosion Behaviors

#### 3.4.1. Macroscopic Corrosion Morphology

**Figure 12:**
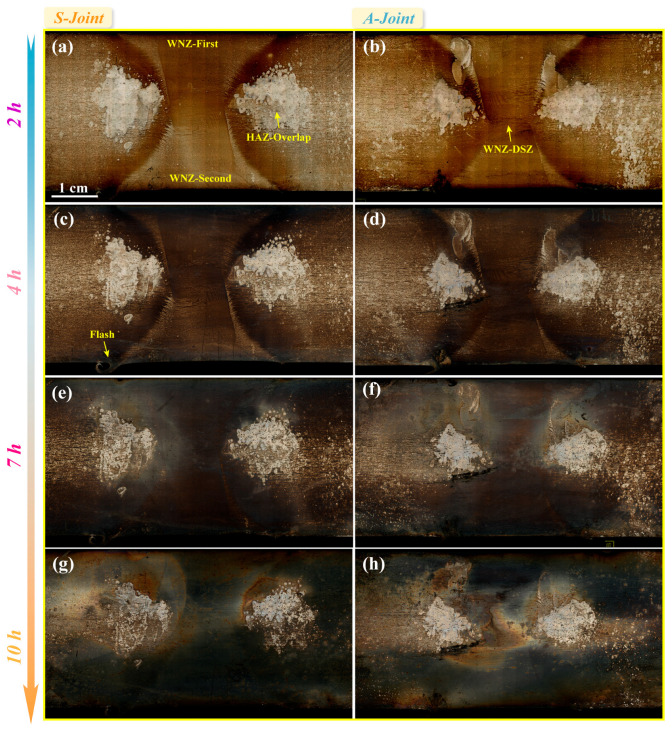
Macroscopic morphologies of S-joint (**a**,**c**,**e**,**g**) and A-joint (**b**,**d**,**f**,**h**) after immersion in 3.5 wt% NaCl solution for 2, 4, 7, and 10 h.

Figure 12 presents the macroscopic micrographs of the weld cross sections for the S-joint and A-joint after immersion in 3.5 wt% NaCl solution for 2, 4, 7, and 10 h. From the earliest stage (2 h of corrosion), the most vulnerable region in both joints is clearly the HAZ-O, which appears as whitish bands on the images (Figure 12a,b). This whitish contrast is attributed to the local breakdown of the passive film, and importantly, the HAZ-O acts as an anode in a macroscopic galvanic couple with the adjacent, more noble WNZs. The large potential difference (up to ~120 mV in the S-joint) drives accelerated anodic dissolution of the HAZ-O, making it the preferential corrosion site. Additionally, the coarse Al-Cu-Fe and Al-Zn-Mg-Cu particles and dense grain-boundary precipitates further intensify micro-galvanic activity within the HAZ-O. For the S-joint, the corroded HAZ area is notably larger than that in the A-joint, consistent with the higher overall heat input from using the same large tool for both passes, leading to more severe precipitate overaging and a broader softened zone. In both joints, the HAZ-O corrosion tends to shift slightly toward the side of the first weld, indicating that the first thermal cycle may induce a more pronounced microstructural degradation than the second pass. As the immersion time increases to 4 h, the HAZ-O whitening intensifies (Figure 12c,d), and by 7 h, macroscopic pits become visible to the naked eye within this region (Figure 12e,f). After 10 h, the number and size of these pits increase substantially, with some pits coalescing into larger cavities (Figure 12g,h).

In contrast, the WNZs exhibit a different behavior. At 2 h and 4 h, the entire WNZ area still retains a metallic luster, suggesting a relatively intact passive film and lower corrosion susceptibility (Figure 12a–d). However, after 7 h, the WNZ surface turns purplish, indicating the formation of a thin corrosion product layer, yet the attack remains much less severe than in the HAZ (Figure 12e,f). This relative protection of the WNZ may originate from its fine recrystallized grain structure and the fragmented, uniformly distributed particles, which promote a more stable passive film and also make the WNZ more cathodic in the macro-galvanic cell. Upon prolonged immersion to 10 h, local pits begin to appear in the WNZ-DSZ, and interestingly, the pit density in the DSZ is significantly higher than that in the WNZ-F or WNZ-S (Figure 12g,h). This preferential pitting of the DSZ, despite its ultra-fine grain size, is speculated to be associated with the extremely high density of Al-Cu-Fe fragments that act as numerous local cathodic sites, thereby facilitating pitting initiation under prolonged exposure. The overall macroscopic evolution indicates that the HAZ-O, driven by macro-galvanic coupling with the WNZs, is the most critical corrosion weak point, with the S-joint suffering a wider attacked area, while the A-joint shows a more confined HAZ-O degradation.

**Figure 13:**
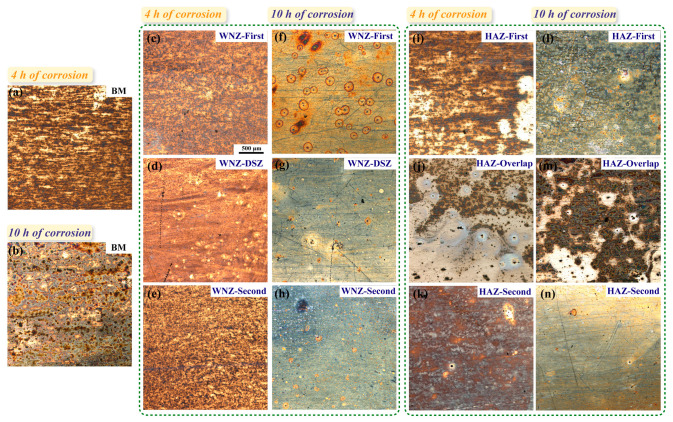
Corrosion morphologies of BM and various zones of S-joint after immersion for 4 and 10 h: (**a**,**b**) BM at 4 h and 10 h; (**c**–**e**) WNZ-F, DSZ, and WNZ-S at 4 h; (**f**–**h**) WNZ-F, DSZ, and WNZ-S at 10 h; (**i**–**k**) HAZ-F, HAZ-O, and HAZ-S at 4 h; (**l**–**n**) HAZ-F, HAZ-O, and HAZ-S at 10 h.

Figure 13 presents the corrosion morphology of the local region for the S-joint after immersion in 3.5 wt% NaCl solution for 4 h and 10 h. The BM (Figure 13a) exhibits relatively uniform corrosion at 4 h, with a thin, evenly distributed oxide layer. After 10 h, extensive pitting is observed across the BM surface, consistent with the progressive breakdown of the passive film and the micro-galvanic action around Fe-rich intermetallic particles (Figure 13b). The WNZs undergo widespread dissolution. At 4 h (Figure 13c–e), the WNZ-F, DSZ, and WNZ-S all show signs of surface roughening, with the WNZ-F displaying slightly more pronounced attack. After 10 h (Figure 13f–h), the WNZ-S turns purplish, indicating a thicker corrosion product layer, whereas the WNZ-F exhibits discrete pitting clusters. The DSZ shows intermediate behavior, with a mixture of general dissolution and local pits.

The most severe corrosion, however, occurs in the heat-affected zones (Figure 13i–k). At 4 h, the HAZ-O already displays dark, deep pits and blackened cavities, while the HAZ-F and HAZ-S show less intense but still notable attack. After 10 h, the HAZ-O pits coalesce into large, interconnected corroded areas, accompanied by significant matrix dissolution (Figure 13m). This preferential degradation of the HAZ-O is attributed to its role as the anodic site in the macro-galvanic couple with the more noble WNZs, combined with its coarse Al-Cu-Fe particles and dense grain-boundary precipitates that promote localized galvanic attack. The extensive dissolution of the WNZ-S, despite its finer grain structure, may be related to the specific thermal history of the second pass, which could produce a less protective passive film.

**Figure 14:**
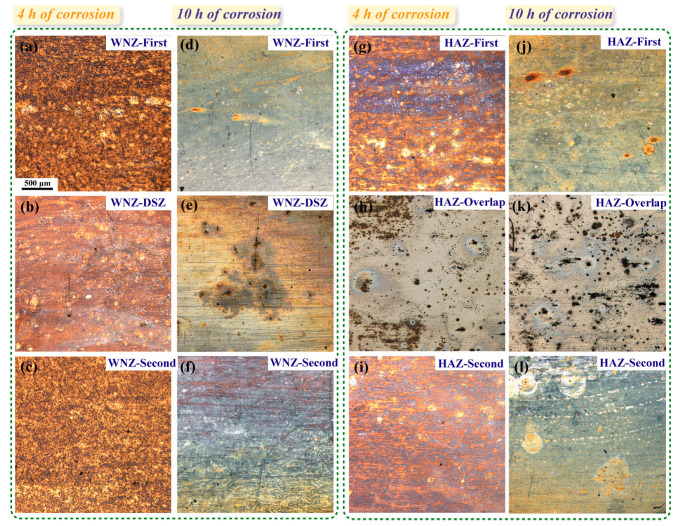
Corrosion morphologies of various zones of A-joint after immersion for 4 and 10 h: (**a**–**c**) WNZ-F, DSZ, and WNZ-S at 4 h; (**d**–**f**) WNZ-F, DSZ, and WNZ-S at 10 h; (**g**–**i**) HAZ-F, HAZ-O, and HAZ-S at 4 h; (**j**–**l**) HAZ-F, HAZ-O, and HAZ-S at 10 h.

For the A-joint, a similar corrosion evolution is observed, albeit with a notably less aggressive attack in the HAZ-O region. After 4 h of immersion, the WNZ-F, DSZ, and WNZ-S (Figure 14a–c) exhibit surface roughening comparable to that of the S-joint, with the WNZ-F again showing slightly more pronounced dissolution. After 10 h (Figure 14d–f), the WNZ-S turns purplish, while the WNZ-F displays discrete pits and the DSZ shows mixed behavior. In the HAZ regions (Figure 14g–i for 4 h; Figure 14j–l for 10 h), the HAZ-F and HAZ-S suffer moderate attack, similar to their S-joint. However, the most distinct difference lies in the HAZ-O. At both 4 h and 10 h, the A-joint exhibits smaller, more isolated pits and less extensive matrix dissolution compared to the S-joint (Figure 14h). The pits in the HAZ-O of the A-joint do not coalesce into large interconnected cavities even after 10 h, indicating a lower corrosion susceptibility (Figure 14k). This milder degradation is attributed to the reduced heat input from the smaller tool (Tool 2) in the second pass, which limits precipitate overaging and narrows the softened zone in the HAZ-O, thereby reducing its anodic activity within the macro-galvanic couple. Overall, while the A-joint follows the same trend of preferential HAZ-O attack, the severity is significantly diminished relative to the S-joint, consistent with the improved electrochemical performance noted earlier.

#### 3.4.2. Microscopic Corrosion Morphology

**Figure 15:**
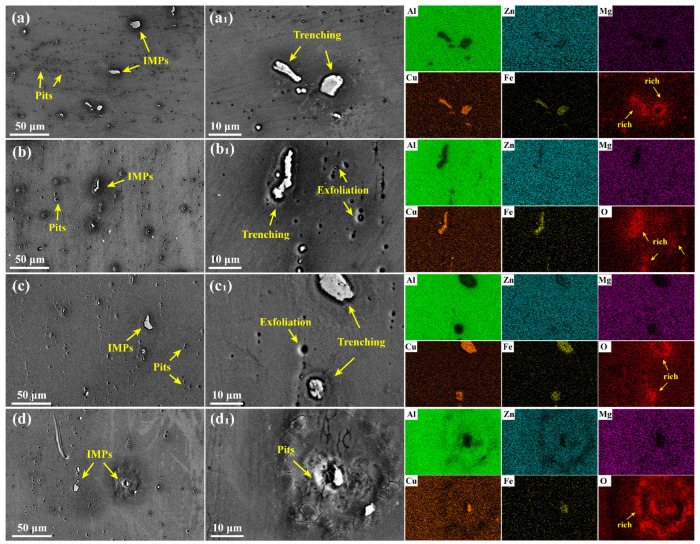
SEM images and EDS mapping of BM after immersion in 3.5 wt% NaCl solution for 2–10 h: (**a**) 2 h; (**b**) 4 h; (**c**) 7 h; (**d**) 10 h. Figures (**a1**–**d1**) are high-magnification images.

Figure 15 presents the SEM images of the BM surface after immersion in 3.5 wt% NaCl solution for 2, 4, 7, and 10 h, with higher-magnification and EDS images shown in Figure 15a1–d1. After 2 h of immersion (Figure 15a), local trenching is clearly observed surrounding the IMPs. These trenches appear as dark halos around the particles, and EDS mapping reveals a significant enrichment of oxygen in these regions, indicating the formation of corrosion products, primarily aluminum hydroxides or oxyhydroxides. The trenching is attributed to micro-galvanic coupling between the noble Fe-rich particles (cathodic) and the adjacent Al matrix (anodic), leading to the preferential dissolution of the matrix around the particles [27]. As the immersion time increases to 4 h (Figure 15b,b1), the trenches expand laterally, and some smaller particles begin to detach from the matrix, leaving behind shallow pits. The oxygen-rich areas become more extensive, suggesting continued anodic dissolution and reprecipitation of corrosion products. After 7 h (Figure 15c,c1), further trench widening and particle detachment are evident, with several pits coalescing into larger cavities. The remaining Fe-rich particles stand proud of the corroded surface, confirming their cathodic nature. By 10 h (Figure 15d,d1), numerous large pits with diameters exceeding several micrometers are observed, often associated with sites where particles have completely fallen out. EDS analysis at these locations shows strong oxygen signals, indicating that the pits are filled with a mixture of residual Al-oxyhydroxide and chloride-containing corrosion products.

**Figure 16:**
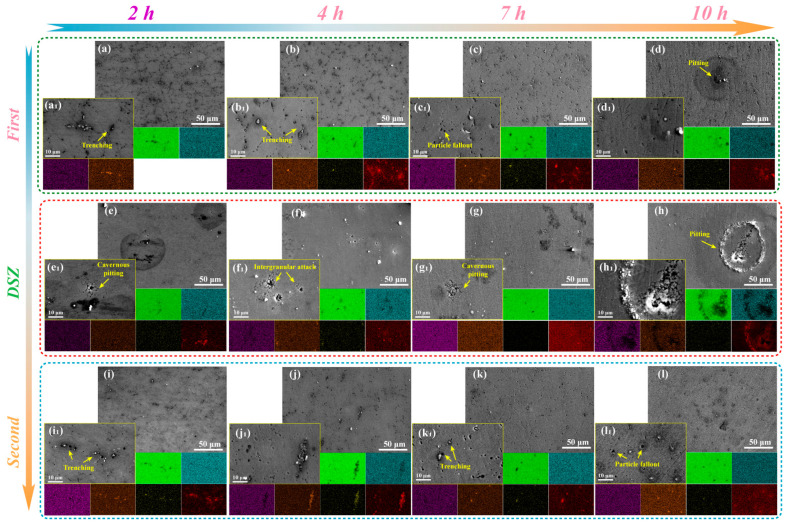
SEM images and EDS mapping of various zones in S-joint after immersion in 3.5 wt% NaCl solution for 2–10 h: (**a**–**d**) WNZ-F at 2, 4, 7, and 10 h; (**e**–**h**) WNZ-DSZ at 2, 4, 7, and 10 h; (**i**–**l**) WNZ-S at 2, 4, 7, and 10 h. Figures (**a1**–**l1**) are high-magnification images.

Figure 16 presents the SEM micrographs of distinct regions for the S-joint after immersion in 3.5 wt% NaCl solution, covering the WNZ-F (a–d), WNZ-DSZ (e–h), and WNZ-S (i–l). After 2 h, all WNZ regions exhibit pronounced trenching around coarse Al-Cu-Fe and Al-Zn-Mg-Cu particles, with EDS revealing significant oxygen enrichment in the trenches (Figure 16a1,i1). By 4 h, the trenches deepen and widen, and some smaller particles begin to loosen (Figure 16b1). At 7 h, particle fallout is clearly observed in the WNZ-F and WNZ-S (Figure 16c1,k1), leaving behind cavernous pits (cavernous pitting) where the matrix around the particle has been completely undermined. The WNZ-DSZ, however, shows a distinctly different attack mode. Instead of particle-induced trenching, the DSZ exhibits intergranular attack propagating along the ultra-fine grain boundaries (Figure 16e1,f1). By 7 h, the intergranular corrosion evolves into ring-shaped pits (annular pitting) as the grain-boundary network is progressively consumed, leading to grain detachment (Figure 16g). This transition from intergranular attack to ring-shaped pitting likely results from the coalescence of multiple boundary dissolution fronts, where the isolated grains fall out, leaving circular cavities. EDS at these sites shows even higher oxygen accumulation, consistent with the formation of voluminous corrosion products. Comparing the two WNZs, the WNZ-S consistently exhibits milder corrosion than the WNZ-F at each time interval. After 10 h, the WNZ-F shows extensive particle fallout and large irregular pits, whereas the WNZ-S retains a more intact surface with fewer and smaller pits (Figure 16d1,l1). This difference is speculated to arise from the lower number density of coarse particles in the WNZ-S, as suggested by the particle distribution analysis (Section 3.2). A reduced cathodic site density diminishes the micro-galvanic driving force, thereby suppressing trenching and pit initiation. The DSZ, despite its finest grain size, suffers the most severe intergranular attack due to the high density of nanoscale boundary precipitates, which create a continuous network of anodic pathways (Figure 16h).

**Figure 17:**
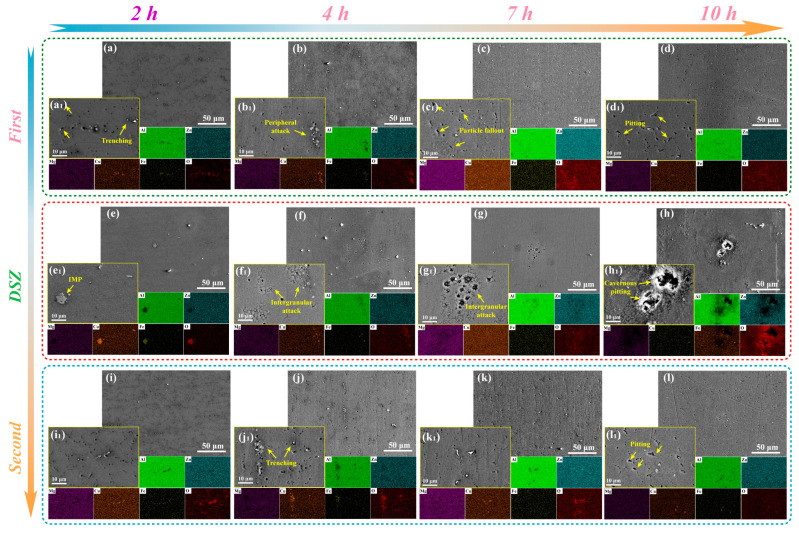
SEM images and EDS mapping of various zones in A-joint after immersion in 3.5 wt% NaCl solution for 2–10 h: (**a**–**d**) WNZ-F at 2, 4, 7, and 10 h; (**e**–**h**) WNZ-DSZ at 2, 4, 7, and 10 h; (**i**–**l**) WNZ-S at 2, 4, 7, and 10 h. Figures (**a1**–**l1**) are high-magnification images.

**Figure 18:**
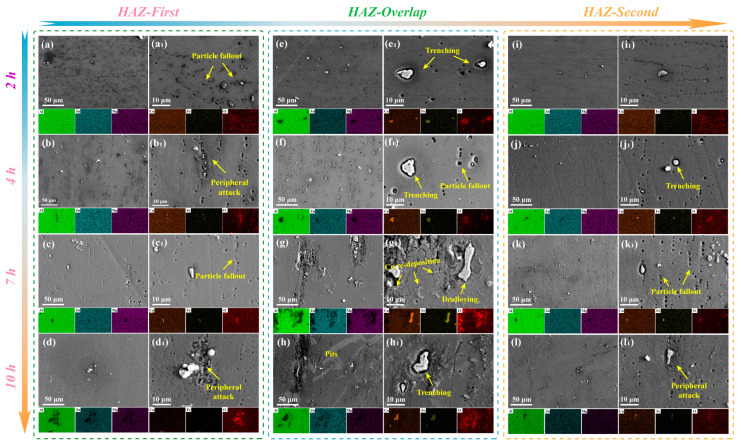
SEM images and EDS mapping of various zones in S-joint after immersion in 3.5 wt% NaCl solution for 2–10 h: (**a**–**d**) HAZ-F at 2, 4, 7, and 10 h; (**e**–**h**) HAZ-O at 2, 4, 7, and 10 h; (**i**–**l**) HAZ-S at 2, 4, 7, and 10 h. Figures (**a1**–**l1**) are high-magnification images.

For the A-joint, a similar time-dependent corrosion evolution is observed in the WNZ-F, WNZ-DSZ, and WNZ-S regions (Figure 17a–l, with magnified views in a1–l1). At 2 h, pronounced trenching around Al-Cu-Fe and Al-Zn-Mg-Cu particles is evident in all WNZs, accompanied by oxygen-rich halos (Figure 17a1,e1,i1). By 4 h, trench widening and particle loosening occur (Figure 18b1,j1). After 7 h, particle fallout and cavernous pitting are visible in the WNZ-F and WNZ-S, though the pits appear slightly smaller and less numerous than in the S-joint (Figure 17e1,k1). The DSZ again exhibits intergranular attack along the ultra-fine grain boundaries, which by 7 h transforms into ring-shaped pitting (Figure 17g). EDS confirms strong oxygen enrichment in these annular pits. Notably, the WNZ-S of the A-joint shows even milder corrosion compared to its Sym counterpart, consistent with its finer and more uniformly distributed particles that reduce local cathodic site density and improve passive film stability. After 10 h, the WNZ-F displays moderate particle fallout and isolated pits, while the WNZ-S retains a relatively intact surface with only minor pitting (Figure 17d1,l1). The DSZ, however, suffers the most severe intergranular attack among the three zones, similar to the Sym case, indicating that the ultra-fine grain structure with boundary precipitates remains the most susceptible to localized corrosion despite the overall improved performance of the A-joint (Figure 17h1).

Figure 18 presents the SEM micrographs of the HAZ regions in the S-joint after immersion in 3.5 wt% NaCl solution for 2, 4, 7, and 10 h. Compared to the WNZs, all HAZ regions exhibit much more pronounced trenching around coarse Al-Cu-Fe and Al-Zn-Mg-Cu particles due to the absence of mechanical fragmentation and the presence of dense grain-boundary precipitates. At 2 h, deep trenches already form around the large particles, with EDS showing intense oxygen enrichment (Figure 18a–d). In the HAZ-F and HAZ-S, peripheral attack characterized by localized dissolution along the particle/matrix interface progresses rapidly, leading to extensive particle fallout by 4–7 h and leaving behind irregular pits (Figure 18b,e,j,h). The HAZ-O, however, shows the most severe degradation. At 2 h, trenching is exceptionally wide and deep, and by 7 h, elongated corrosion cavities exceeding 70 µm in length develop, often aligned with the elongated grain structure or precipitate stringers (Figure 18e–g). Notably, some large Al-Cu-Fe particles in the HAZ-O undergo dealloying, as evidenced by a flocculent, porous surface morphology on the particles themselves (Figure 18g). This dealloying process selectively dissolves the more active Al elements from the particle, leaving behind a Cu-rich residue. Concurrently, fine Cu-rich spots (tens to hundreds of nanometers) are observed in the surrounding matrix, indicating redeposition of dissolved copper ions via a local galvanic mechanism. The dealloyed particle acts as a cathodic site, promoting the further anodic dissolution of the adjacent matrix, and Cu^2+^ ions from the particle reduction deposit as metallic Cu on nearby surfaces [35]. After 10 h, the HAZ-O exhibits massive, interconnected pits with extensive matrix loss, while the HAZ-F and HAZ-S show widespread particle fallout and coarsened pits, but these are less severe than in the overlap region (Figure 18d,h,l). The aggressive attack in the HAZ-O is attributed to the combined effects of the highest density of coarse Al-Cu-Fe particles, severe grain-boundary precipitation from dual thermal cycles, and the HAZ-O’s role as the anodic site in the macro-galvanic couple with adjacent WNZs. Notably, in the S-joint HAZ-O, the corrosion products appear porous and poorly adherent, with evidence of spallation (detachment) from the surface, exposing fresh metal to the electrolyte. This is consistent with the severe macro-galvanic attack and high corrosion current density observed in this region.

**Figure 19:**
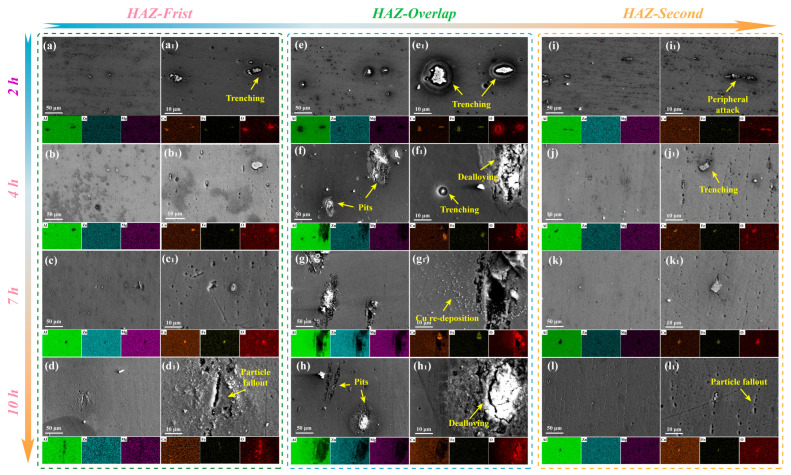
SEM images and EDS mapping of various zones in A-joint after immersion in 3.5 wt% NaCl solution for 2–10 h: (**a**–**d**) HAZ-F at 2, 4, 7, and 10 h; (**e**–**h**) HAZ-O at 2, 4, 7, and 10 h; (**i**–**l**) HAZ-S at 2, 4, 7, and 10 h. Figures (**a1**–**l1**) are high-magnification images.

For the A-joint, as shown in Figure 19, in the HAZ-F, extensive trenching encircles the coarse Al-Cu-Fe particles from 2 h onward, and by 4–10 h, numerous particles have detached, leaving behind pits (Figure 19a–d). The HAZ-O exhibits the most intense attack. At 7 h and 10 h, large particles show signs of dealloying, with their surfaces becoming flocculent and porous. Surrounding areas display fine, Cu-rich speckles, indicating the local redistribution of Cu (Figure 19e–h). The HAZ-S shows milder corrosion, characterized by isolated particle detachment and limited trenching (Figure 19i–l). This reduced susceptibility is attributed to the lower heat input from the smaller tool, which lessens precipitate coarsening along grain boundaries and decreases the density of active micro-galvanic cells, thereby suppressing trench propagation.

## 4. Discussion

### 4.1. Corrosion Mechanism of DS-FSW Joints

The corrosion behavior of DS-FSW AA7A65 joints is governed by a hierarchical galvanic coupling system, including macroscopic galvanic corrosion between different weld zones (dominant) and microscopic galvanic corrosion induced by IMPs. The interplay of these two mechanisms, modulated by the welding configuration (symmetric vs. asymmetric), determines the overall corrosion resistance.

For macro-galvanic corrosion (the primary driver): The OCP and PDP measurements clearly establish a well-defined galvanic series across the weld cross section. The potential difference between the BM and HAZ-O reaches ~120 mV in the S-joint, creating a strong macro-galvanic cell where the HAZ-O acts as the primary anode and the BM/WNZs serve as cathodes. This macro-galvanic effect is the principal reason why the HAZ-O consistently appears as the most severely corroded region. The electron flow from the anodic HAZ-O through the metallic matrix to the cathodic WNZ drives oxygen reduction at the cathode surface, accelerating the anodic dissolution of the HAZ-O. This is consistent with the findings of [35], which reported that macro-galvanic effects dominate the corrosion of FSW aluminum alloy weldments [36]. In the A-joint, the potential spread is reduced to ~74 mV, directly correlating with the less aggressive HAZ-O attack.

For micro-galvanic corrosion (local initiation and propagation): At the microscopic level, Al-Cu-Fe and Al-Zn-Mg-Cu particles act as local cathodes relative to the Al matrix [37]. The Volta potential difference between Cu-rich IMPs and the Al matrix can reach +200–400 mV [38], inducing trenching around the particles. This effect is universal but varies in intensity across different zones. In the HAZs, coarse IMPs (10–30 µm) provide large cathodic surface areas that drive intense trenching. In the WNZs, fragmentation of IMPs reduces the local cathodic current density per particle, explaining the milder micro-galvanic attack in the WNZ-S of the A-joint.

#### 4.1.1. For Macro-Galvanic Corrosion: The Primary Driver

The OCP and PDP measurements clearly establish a well-defined galvanic series across the weld cross section. For the S-joint, the BM exhibits the noblest OCP and the lowest corrosion current density, followed by the WNZs. The HAZs are significantly more active, with the HAZ-O showing the most negative potential and the highest *i_corr_*. The potential difference between the BM and HAZ-O reaches ~120 mV, creating a strong macro-galvanic cell where the HAZ-O acts as the primary anode and the BM/WNZs serve as cathodes [39]. This macro-galvanic effect is the principal reason why the HAZ-O consistently appears as the most severely corroded region in macroscopic immersion images. In contrast, the A-joint displays a much narrower potential spread, and the potential difference between the HAZ-O and WNZ-S is only about 74 mV (compared to ~85 mV in the S-joint). This reduction in the galvanic driving force directly correlates with the less aggressive HAZ-O attack observed in the A-joint (smaller pits and less matrix dissolution).

The macro-galvanic current density can be estimated from the mixed-potential theory. The larger the difference in the OCP and the smaller the polarization resistance of the anode, the higher the galvanic current. The EIS results support this. The HAZ-O in the S-joint exhibits an extremely low film resistance, whereas in the A-joint, the corresponding |*Z*|*_mod_* is even lower, yet the galvanic driving force is smaller, resulting in net less attack [31,32]. This apparent paradox is resolved by considering that the Asy HAZ-O, although it has a very low impedance, is less effectively coupled to a large cathodic area because the WNZs themselves are also less noble. The overall corrosion rate is thus controlled by both the anode’s intrinsic susceptibility and the macro-galvanic coupling strength.

The corrosion of the aluminum matrix occurs via an electrochemical reaction (Al → Al^3+^ + 3e^−^) at the metal/electrolyte interface. This is an activation-controlled (charge transfer-controlled) process, as evidenced by the capacitive loops in the EIS Nyquist plots (Figure 9a,d), rather than a diffusion-controlled process. The absence of a Warburg element in the EIS spectra (except for the BM) confirms that the solid-state diffusion of alloying elements does not play a significant role in the short-term corrosion behavior studied here [40]. The corrosion propagates inward as “trenching” around IMPs or as pitting, driven by the electrochemical potential difference between the Al matrix and the cathodic Cu/Fe-rich IMPs [41].

#### 4.1.2. Micro-Galvanic Corrosion: Local Initiation and Propagation

At the microscopic level, Al-Cu-Fe and Al-Zn-Mg-Cu particles act as local cathodes relative to the Al matrix, inducing trenching around the particles. This effect is universal but varies in intensity across different zones [27]. In the HAZs, IMPs remain coarse (10–30 µm), often clustered along grain boundaries, and are surrounded by precipitate-depleted zones. The large cathodic surface area of these particles drives intense trenching. After only 2 h, deep oxygen-rich trenches form around the particles, and by 4–7 h, particle fallout leaves cavernous pits (as seen in the SEM images). In the HAZ-O of the S-joint, some large IMPs even undergo dealloying (selective dissolution of the Al element), leaving behind a porous Cu-rich skeleton [42]. Subsequently, fine Cu-rich redeposits appear on the adjacent matrix, further enhancing local cathodic activity. This micro-galvanic process accelerates the macro-galvanic attack on the HAZ-O.

In the WNZs, IMPs are fragmented by the severe plastic deformation during FSW. In the S-joint, the WNZ-F and WNZ-S still contain some coarse fragments (15–30 µm) that cause trenching and particle fallout. However, the WNZ-S in the A-joint exhibits exceptionally fine (2–3 µm), rounded, and uniformly dispersed IMPs, which dramatically reduce the local cathodic current density per particle. This explains why the WNZ-S of the A-joint shows the mildest micro-galvanic trenching and the highest impedance among all welded zones. The DSZ presents a unique case. Despite having ultra-fine grains (down to the nanoscale in some regions) and fragmented IMPs, it suffers from intergranular attack rather than isolated pitting. High-magnification SEM reveals that the DSZ contains a high density of nanoscale Al-Cu-Fe and Al-Zn-Mg-Cu fragments decorating the grain boundaries. These boundary-decorating precipitates create a continuous network of local cathodes, promoting preferential dissolution along the grain boundaries. This intergranular corrosion evolves into ring-shaped pits (annular pitting) as entire grains fall out [43]. The DSZ in the S-joint shows more severe intergranular attack than that in the A-joint, consistent with the latter’s more uniform particle distribution.

### 4.2. Comparison of Corrosion Resistance of S-Joint and A-Joint

The superior corrosion resistance of the A-joint can be traced to three microstructural modifications induced by the asymmetric welding process:

#### 4.2.1. Grain Size Refinement in the Second-Pass WNZ and HAZ-S

The smaller tool generates a lower heat input and higher strain rate, producing a finer recrystallized grain structure in the lower weld. Fine grains promote the formation of a more homogeneous and protective passive film, as evidenced by the higher |Z| values and lower *i_corr_*.

#### 4.2.2. IMP Fragmentation and Spheroidization

In the Asy WNZ-S, the Al-Cu-Fe and Al-Zn-Mg-Cu particles are broken down to 2–3 µm and adopt rounded morphologies, which reduces the cathodic surface area and mitigates micro-galvanic trenching. In contrast, the WNZ-S of the S-joint still contains angular, blocky particles up to 15–30 µm that act as efficient cathodes.

#### 4.2.3. Less Severe HAZ Degradation

The lower heat input of Tool 2 limits the overaging of grain-boundary precipitates in the HAZ-S and HAZ-O. Consequently, the HAZ-O in the A-joint, while still the weakest region, exhibits less extensive dealloying, fewer Cu-rich deposits, and smaller pits compared to the S-joint. The narrower potential spread (reduced macro-galvanic driving force) further protects the HAZ-O.

The superior corrosion resistance of the A-joint can be traced to three microstructural modifications induced by the asymmetric welding process, as quantitatively summarized in Table 4. First, grain refinement in the second-pass WNZ and HAZ-S (8–15 µm vs. 10–20 µm in the S-joint) promotes the formation of a more homogeneous and protective passive film. Second, the effective fragmentation and spheroidization of IMPs (8–20 µm, rounded vs. 10–30 µm, angular) reduce the local cathodic site density, mitigating micro-galvanic trenching. Third, less severe grain-boundary precipitation in the HAZ-S reduces the number of active anodic sites along grain boundaries. In summary, the S-joint suffers from severe macro-galvanic corrosion driven by a large potential difference between the highly anodic HAZ-O and the noble BM/WNZs. This corrosion is further exacerbated by coarse, poorly fragmented IMPs that promote intense micro-galvanic trenching and dealloying. The A-joint uses a smaller tool for the second pass, achieving a more homogeneous electrochemical landscape (smaller potential gradients), refining the IMPs, and preserving the HAZ microstructure. These improvements thereby mitigate both macro- and micro-galvanic attacks. These mechanistic insights demonstrate that asymmetric DS-FSW is an effective strategy to enhance the corrosion durability of thick-plate AA7A65 welds.

#### 4.2.4. Role of Passive Film Stability and Electrode Composition

It is important to recognize that the corrosion resistance of DS-FSW joints is not determined solely by the passive film itself but rather by the interplay between the passive film stability and local electrode composition. In the S-joint, the coarse, angular Al-Cu-Fe and Al-Zn-Mg-Cu particles (15–30 µm) act as efficient cathodes, driving the intense micro-galvanic corrosion of the surrounding Al matrix. The anodic dissolution of the matrix leads to localized acidification (due to Al^3+^ hydrolysis) and the formation of porous, poorly adherent corrosion products (Al hydroxides/oxyhydroxides). These products are prone to spallation (detachment) under immersion, exposing fresh metal to the electrolyte and perpetuating the corrosion cycle [39]. Furthermore, the dealloying of Al-Cu-Fe particles where Al is selectively dissolved, leaving behind a Cu-rich porous skeleton, alters the local electrode composition, making the remaining Cu-rich residue even more cathodic and further destabilizing the passive film [2,44]. In contrast, the A-joint, with its finer, rounded IMPs (2–3 µm in the WNZ-S; 8–20 µm in the HAZ-S), promotes the formation of a denser, more adherent passive film, as evidenced by the absence of a low-frequency inductive loop in the EIS spectra. The reduced macro-galvanic driving force (~74 mV potential spread) also minimizes the local pH changes and hydrogen evolution that would otherwise promote passive film breakdown [45].

## 5. Conclusions

This study systematically investigated the corrosion behavior of S-joint and A-joint double-sided friction stir-welded AA7A65 aluminum alloy thick plates in 3.5 wt% NaCl solution, focusing on the microstructure–electrochemical corrosion–immersion corrosion relationship. The principal findings are summarized as follows:

(1) The S-joint, welded with the same large tool (Φ30 mm) for both passes, exhibits symmetric but insufficiently fragmented IMPs, coarse recrystallized grains in the WNZ, and severely overaged HAZs with dense grain-boundary precipitates. In contrast, the A-joint, employing a smaller tool (Φ24 mm) for the second pass, achieves an asymmetric microstructure: the WNZ-S contains fine, rounded, uniformly dispersed IMPs (2–3 µm), while the double-stirred zone exhibits ultra-fine grains (~2 µm) and the HAZ-S shows less thermal degradation (grain size: 8–15 µm vs. 10–20 µm in the S-joint).
(2) Electrochemical measurements reveal that the HAZ-O is the most anodic zone in both joints. The S-joint exhibits a larger potential spread (up to ~120 mV) and higher corrosion current density (HAZ-O: 7.28 μA·cm^−2^) than the A-joint (HAZ-O: 5.89 μA·cm^−2^). Electrochemical impedance spectroscopy shows that the A-joint second-pass WNZ and HAZ lack a low-frequency inductive loop and display significantly higher impedance (WNZ-S: 41,412 Ω·cm^2^ vs. 21,664 Ω·cm^2^ in the S-joint), indicating superior passive film stability.
(3) Immersion tests demonstrate that macro-galvanic coupling between the anodic HAZ-O and the cathodic weld nuggets drives severe localized attack. The S-joint suffers wider and deeper HAZ-O corrosion, with pit coalescence into large cavities after 10 h. The A-joint mitigates this damage, showing smaller, more isolated pits. Microscopically, the HAZ-O exhibits dealloying of coarse IMPs and Cu-rich redeposition, while the double-stirred zone undergoes intergranular attack evolving into annular pitting due to nanoscale IMPs decorating grain boundaries.
(4) The superior corrosion resistance of the A-joint is attributed to three microstructural modifications induced by the asymmetric process: (i) grain refinement in the second-pass WNZ and HAZ (8–15 µm), promoting a more protective passive film; (ii) the effective fragmentation and spheroidization of IMPs (2–3 µm in the WNZ-S; 8–20 µm in the HAZ-S), reducing the local cathodic site density; and (iii) less severe HAZ degradation with narrower potential spread (~74 mV vs. ~85 mV), thereby reducing both macro- and micro-galvanic driving forces.
(5) This study elucidates the mechanisms of macro- and micro-galvanic corrosion in double-sided friction stir-welded AA7A65 joints. The asymmetric DS-FSW strategy, by employing a smaller tool for the second pass, effectively enhances the corrosion durability of thick-plate aluminum alloy joints, providing a valuable microstructural design guideline for aerospace applications.

## Figures and Tables

**Table 1:** Nominal chemical composition of 7A65 aluminum alloy (wt%).

Al	Zn	Mg	Cu	Fe	Si	Mn	Ti	Zr	Cr
Bal.	7.70	1.65	1.00	0.08	0.06	0.04	0.06	0.10	0.04

**Table 2:** The chemical compositions (at.%) of the marked positions (A–L) corresponding to the IMPs of the WNZs in the S-joint and A-joint.

	A	B	C	D	E	F	G	H	I	J	K	L
Al	76.59	87.87	74.25	91.57	73.42	59.80	74.83	71.22	89.25	82.54	79.71	80.79
Zn	2.36	5.59	2.26	4.57	1.91	13.99	2.11	1.82	5.40	3.78	3.36	7.80
Mg	0.77	3.98	0.73	2.42	0.57	15.66	0.62	0.55	3.39	1.67	1.32	6.89
Cu	14.00	2.51	15.49	1.44	16.32	10.51	15.43	18.08	1.95	7.91	10.44	4.49
Fe	6.28	0.05	7.27	0.00	7.78	0.05	7.01	8.35	0.02	4.10	5.17	0.03

**Table 3:** The chemical compositions (at.%) of the marked positions (A–L) corresponding to the IMPs of the HAZs in the S-joint and A-joint.

	A	B	C	D	E	F	G	H	I	J	K	L
Al	75.24	73.08	69.20	78.81	71.79	75.44	70.84	80.86	68.75	70.27	75.81	68.49
Zn	2.17	2.04	1.12	3.11	1.93	1.99	1.33	4.01	0.77	1.15	2.45	0.74
Mg	0.95	0.79	0.06	1.25	0.60	0.69	0.37	1.78	0.13	0.31	0.65	0.23
Cu	14.97	16.50	0.29	11.53	18.31	14.95	18.87	9.49	21.05	19.52	14.30	20.94
Fe	6.66	7.60	9.44	5.31	7.37	0.23	8.59	3.86	9.29	8.75	6.79	9.61

**Table 4:** A comparison of the grain size and IMP characteristics in the HAZ regions of the S-joint and A-joint.

Zone	Joint	Grain Size (μm)	IMP Size (μm)	IMP Morphology	Grain-Boundary Precipitation
HAZ-F	S	10–25	10–30	Angular, blocky, clustered	Moderate to dense
	A	10–25	10–30	Angular, blocky	Moderate
HAZ-A	S	10–20	10–30	Angular, poorly fragmented	Moderate
	A	8–15	8–20	Finer, more rounded	Reduced
HAZ-O	S	8–18	15–35	Coarse, angular, dealloying	Most dense
	A	8–18	15–35	Coarse, heterogeneous	Dense, but less severe

**Table 5:** Average OCP values (V vs. Ag/AgCl) of BM and different zones of S-joint and A-joint.

	WNZ-F	WNZ-D	WNZ-S	HAZ-F	HAZ-O	HAZ-S	BM
S-joint	−0.712	−0.719	−0.706	−0.766	−0.791	−0.750	−0.671
A-joint	−0.692	−0.680	−0.695	−0.731	−0.754	−0.720	

**Table 6:** The *E_corr_* and *i_corr_* values of the BM and different microstructural zones of the S-joint and A-joint derived from Tafel extrapolation.

		WNZ-F	WNZ-D	WNZ-S	HAZ-F	HAZ-O	HAZ-S	BM
*E_corr_* (mV_Ag/AgCl_)	S-joint	−0.682	−0.694	−0.676	−0.746	−0.776	−0.723	−0.652
	A-joint	−0.687	−0.701	−0.678	−0.744	−0.765	−0.722	
*I_corr_* (μA/cm^2^)	S-joint	1.98	1.74	1.45	5.02	7.28	4.41	0.776
	A-joint	1.79	1.52	1.36	4.47	5.89	2.56	

**Table 7:** The fitting results of the EIS data for the S-joint.

Sample	R_S_ (Ω cm^2^)	CPE_f_ (10^−5^·Ω^−1^ cm^−2^·sn)	n_f_	R_f_ (Ω cm^2^)	CPE_dl_ (10^−5^·Ω^−1^ cm^−2^·sn)	n_dl_	R_ct_ (Ω cm^2^)	L (H·cm^2^)	R_l_ (Ω cm^2^)
BM	11.2	82.94	0.68	4811	56.55	0.60	18,140	-	-
WNZ-F	12.0	39.10	0.77	4638	27.31	0.69	3563	10,850	1425
WNZ-D	17.6	43.51	0.79	1864	38.32	0.85	6425	8087	2547
WNZ-S	11.8	26.44	0.75	3815	19.56	0.66	5879	11,240	1875
HAZ-F	24.2	19.56	0.89	2161	60.18	0.81	10,950	3875	1452
HAZ-O	24.3	11.88	0.95	2716	3.71	0.94	48,280	8338	880
HAZ-S	23.6	30.97	0.85	1681	30.52	0.24	12,801	2187	2054

**Table 8:** The fitting results of the EIS data for the A-joint.

Sample	R_S_ (Ω cm^2^)	CPE_f_ (10^−5^·Ω^−1^ cm^−2^·sn)	n_f_	R_f_ (Ω cm^2^)	CPE_dl_ (10^−5^·Ω^−1^ cm^−2^·sn)	n_dl_	R_ct_ (Ω cm^2^)	L (H·cm^2^)	R_l_ (Ω cm^2^)
WNZ-F	24.9	11.41	0.92	1841	26.7	0.68	5930	13,510	6587
WNZ-D	22.7	30.36	0.82	6372	34.7	0.52	3242	-	-
WNZ-S	23.4	13.37	0.91	8750	18.5	0.81	7513	-	-
HAZ-F	22.7	19.63	0.92	3016	51.3	0.69	10,250	18,940	2632
HAZ-O	22.5	15.08	0.90	2038	21.2	0.74	4901	19,530	969.4
HAZ-S	23.6	37.85	0.82	2585	66.1	0.74	9072	-	-

## Data Availability

The original contributions presented in this study are included in the article. Further inquiries can be directed to the corresponding author.
